# Supplementary material for: Ixodes scapularis microbiome correlates with life stage, not the presence of human pathogens, in ticks submitted for diagnostic testing
Source: PeerJ. 2020 Dec 2;8:e10424. doi: 10.7717/peerj.10424 (PMC7718787; doi:10.7717/peerj.10424)
Supplement: Supplemental Information 5 — Shannon diversity of the adults and nymphs, significance (p) was determined by Kruskal–Wallis test. ASVs below a given percentage of the sum were removed to test for inflation of alpha diversity caused by low abundant taxa. Statistically significant p-values (p < 0.05) are in bold. [file peerj-08-10424-s005.docx]

| ASV percent of the sum removed | X² | *p* |
| --- | --- | --- |
| < 0.1% | 11.458 | **0.0007177** |
| < 1% | 8.0391 | **0.004578** |
| < 5% | 3.1566 | 0.07562 |
| < 10% | 2.5786 | 0.1083 |
