# Supplementary figures and images for: Ixodes scapularis microbiome correlates with life stage, not the presence of human pathogens, in ticks submitted for diagnostic testing
Source: PeerJ. 2020 Dec 2;8:e10424. doi: 10.7717/peerj.10424 (PMC7718787; doi:10.7717/peerj.10424)

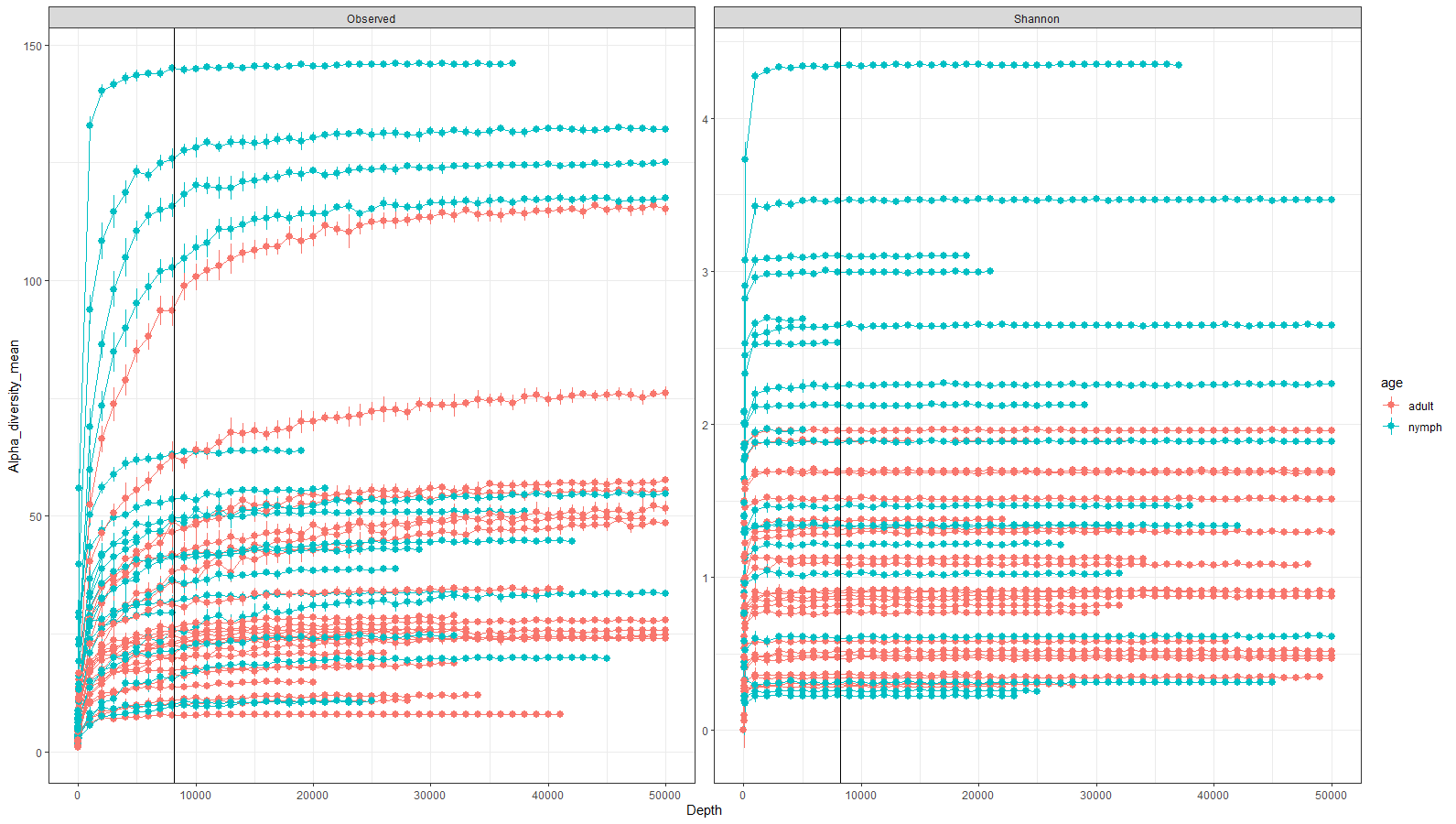

Supplement: Supplemental Information 8 — Rarefaction curves showing mean alpha diversity of observed ASVs and Shannon diversity index. Means were calculated at depths of 1, 10, 100, 1,000 and every 1,000 reads (as shown by each data point). Adults shown in (red) and nymphs in (blue). A black line is added at a depth of 8200 reads. Only two samples failed to have a sampling depth of 8200 reads. [file peerj-08-10424-s008.png]

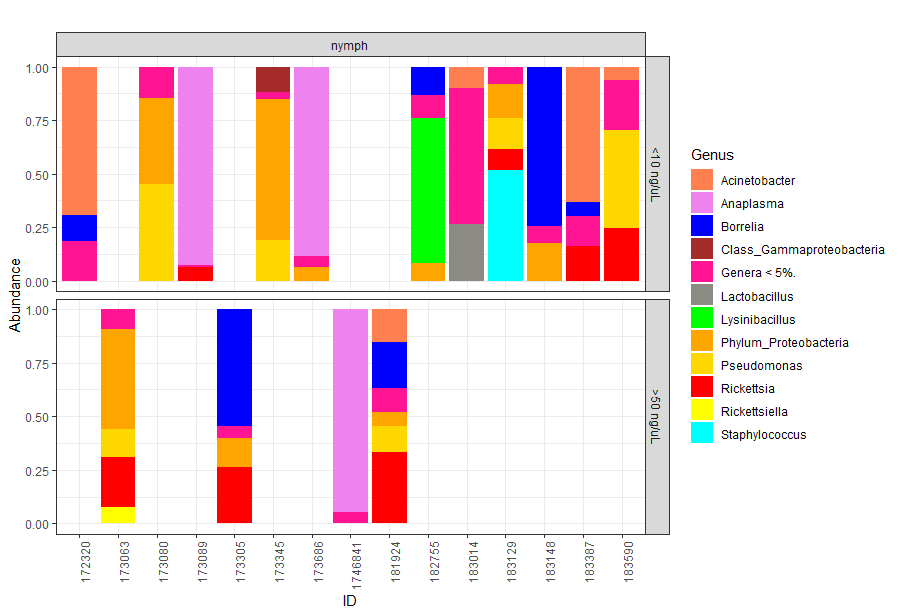

Supplement: Supplemental Information 9 — Relative abundance of genera at low ( <10 ng/ μL) post extraction DNA concentrations (low biomass) and high ( >50 ng/ μL) concentration of DNA post extraction (large biomass). High levels of variability are still observed in large biomass samples, comparable to those observed in the low biomass samples. Taxa that comprised less than 5% of each sample were regrouped to a new category [file peerj-08-10424-s009.png]

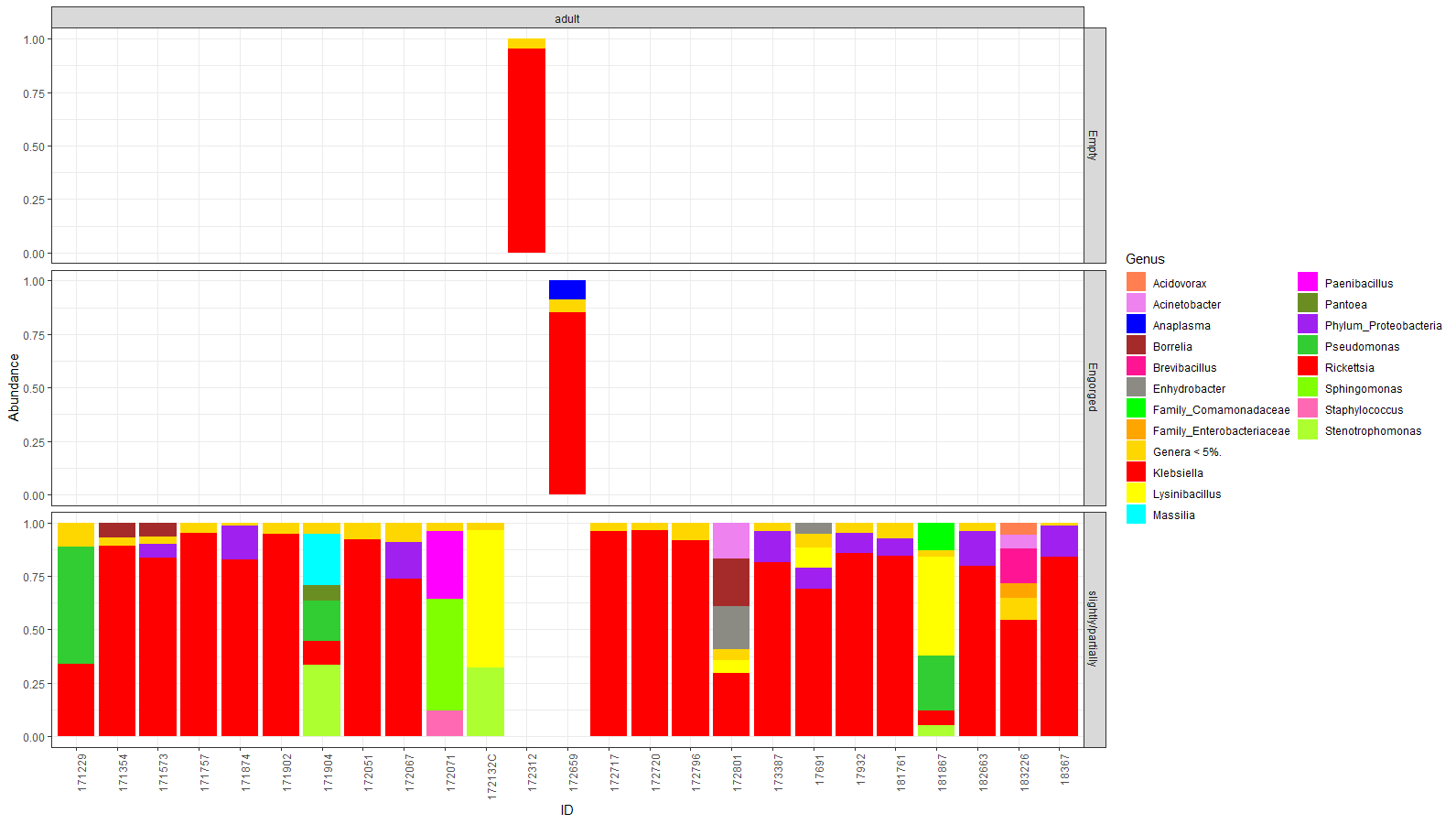

Supplement: Supplemental Information 10 — Adults were classified as either empty/non-engorged ( n = 1), engorged ( n = 1), or slightly/partially ( n = 23). Non-engorged and engorged were comparable to the majority of slightly/partially engorged ticks. [file peerj-08-10424-s010.png]
